# The Relationship between Weight Bias Internalization and Quality of Life among Overweight and Obese Youths in Thailand

**DOI:** 10.4314/ejhs.v34i3.3

**Published:** 2024-05

**Authors:** Suneerat Yangyuen, Supattra Keawmuang, Atchara Chaichan

**Affiliations:** 1 Faculty of Public Health, Mahasarakham University, Thailand; 2 Faculty of Nursing, Mahasarakham University, Thailand

**Keywords:** Weight bias internalization, quality of life, obese, youths, BMI

## Abstract

**Background:**

Youth with overweight and obesity are susceptible to weight bias internalization (WBI) and tend to experience impaired quality of life (QOL). However, the evidence regarding the relationship between WBI and QOL remains scarce among Thai youth. Thus, this study aimed to assess the association between WBI and QOL among Thai youth with overweight and obesity.

**Methods:**

A cross-sectional design was conducted with 667 university youths with overweight and obesity from northeastern Thailand selected by a multistage sampling method. A self-reported questionnaire was used for data collection. Multivariable logistic regression was applied to examine the association between WBI and QOL.

**Results:**

More than half of the youths (51.4%) were females with a mean body mass index (BMI) of 26.5 kg/m^2^ (SD = 2.5). More than one third (37.8%) of the participants had a high level of WBI, and 48.9% reported being dissatisfied with their body image. Our results indicate higher BMI and greater WBI, and body image dissatisfactions were strongly associated with worse QOL overall and across all domains (physical health, psychological health, social relationships, and environment) after adjusting for all covariates.

**Conclusions:**

WBI, BMI, and body dissatisfaction play an important role in impaired QOL. Thus, the development of intervention strategies or programs should consider the reduction of these factors as a key component of care or treatment for youth with overweight and obesity to improve QOL.

## Introduction

Overweight and obesity in adolescents are recognized as major public health problems worldwide (1). Adolescents with overweight are prone to a greater risk of weight-related health problems, particularly psychological impacts (e.g., depression, stress, and low self-esteem) and impaired QOL (1,2). QOL refers to an individual's subjective assessment of their physical and psychological health and functional performance status (3). However, impaired QOL is not solely due to overweight or obesity.

Other psychological factors may cause QOL impairment in adolescents, such as depression, anxiety, and perceived stigma (2,4), including weight bias internalization (WBI) (4,5).

WBI occurs when individuals who are overweight or obese accept and endorse stereotypes, prejudice, and negative weight-based societal perceptions, leading to self-devaluation due to weight (4,6). WBI has been associated with negative physical and psychological health outcomes, such as decreased physical activity, eating disorders, depression, low self-esteem, body dissatisfaction (BD), and poorer QOL (4-6). The association between WBI and QOL suggests that individuals with overweight or obesity who experience WBI also reported low levels of psychological, physical, social, and environmental QOL (5,7). Moreover, a recent meta-analyses showed that weight stigma was significantly associated with poor psychological QOL among youth and adults (6,8).

In Asia, previous studies indicated that WBI negatively affects both physical and psychological health among children and adolescents (9-12). For example, a study in Hong Kong (9) showed that WBI is associated with reduced physical activity and has a mediating effect on the relationship between physical activity and QOL. Other studies done in Saudi Arabia (12), and China (13) indicated that individuals with overweight or obesity and high WBI were more likely to report unhealthy dietary habits and poorer quality of food than individuals with normal weight. Further, impaired psychological outcomes of WBI have been documented in studies conducted in Iran (11), and Saudi Arabia (12), which reported that adolescents with overweight or obesity had a high WBI, which can lead to psychological distress due to depression, stress, and impaired QOL. In particular, the impact of WBI on QOL was also found in Hong Kong (10), and Iran (11), indicating that adolescents with overweight or obesity and those who experience WBI had low levels of psychological and physical QOL. Thus, WBI is an important challenge facing youth with overweight and obesity, as it may negatively impact their QOL (2).

In Thailand, previous research revealed that 48.2% of adolescents experienced cyberbullying about their weight (14). Additionally, the social trend that a slim figure is the ideal body type has become popular among Thai youth, as they perceive beauty standards based on cultural ideals, placing youth with overweight or obesity at a high risk of psychological distress and impaired QOL (14,15). Hence, Thai youth may encounter WBI and its adverse psychological outcomes if they perceive themselves to be overweight. Although the relationship between WBI and QOL has been reported in other countries (5,7,9,11), evidence suggests that the impact of adolescent obesity on QOL is influenced by cultural contexts, such as cultural differences in attitudes and stigma toward obesity and social expectations for body size (2,4,12). Moreover, most of the studies investigating WBI and QOL were based on individuals actively seeking treatment (5,7). Nevertheless, little research exists on WBI, and evidence concerning the effects of WBI on various domains of QOL and overall QOL among Thai youths with overweight or obesity remains limited (14,15). Given the difference in cultural context, more research is needed to explore this issue in Thailand. Thus, this study aimed to explore the association between WBI and QOL in a community-based sample of university students to better understand how WBI influences QOL. Ultimately, such insight will be useful in developing interventions to mitigate WBI and improve QOL among youth with overweight and obesity.

## Materials and Methods

**Study setting, design, and population**: A cross-sectional study was conducted from October 2022 to November 2023 at three universities located in three regions of northeastern Thailand. The eligible participants were (a) undergraduate students aged 18–22 years with overweight (body mass index [BMI] = 23–24.9 kg/m^2^) or obesity (BMI ≥25 kg/m^2^) as classified by the Asia-Pacific BMI classification (16), (b) no reported communication or mental health problems, and (c) willing to participate. Individuals who provided incomplete questionnaires were excluded from the analysis.

**Sample size and sampling procedure**: The sample size was calculated using Cochran's formula (17) with an estimator of the percentage of adolescents who experienced weight stigma (48.2%) reported by Thumronglaohapun et al. (14). Based on a 95% confidence interval and precision of 4%, the minimum sample size was 600 students plus 10% for non-response adjustment, which equaled 667 students. All students who met the eligible criteria were enrolled using the multistage sampling method. First, we used the lottery method to select three universities. We selected one university from each of the three regions. Second, six faculties from each university were selected at random from a list of faculties at each university. Third, students were selected by systematic random sampling of each university. Every third student reported as being overweight or obese was selected as a participant. If a student was absent or unwilling to participate, the student next on the list was contacted for participation.

**Data collection instrument and data collection procedure**: The data were collected using a self-reported questionnaire developed based on a literature review to collect information concerning sociodemographic factors, WBI, and QOL. Anthropometric measurements were taken using a portable height and weight meter. All procedures for anthropometry measurements were standardized and all assistants were trained accordingly using a prepared field manual for data collection.

**Measurements**: This self-reported questionnaire was composed of three parts as outlined below.

### Predictors

*Socio-demographic factors*: sex, age, monthly household income, and body image satisfaction; all variables were categorized as dichotomous variables. Body image satisfaction was captured by the question, “Are you satisfied with your figure?” (18). The respondents were categorized into two groups: satisfied if they answered “yes” and dissatisfied if they answered “no.” Portable height and weight meters were used to measure the students' height and weight. BMI was calculated as weight (kg) divided by height (m^2^). Overweight and obesity were classified by the Asia-Pacific BMI classification (16) as having a BMI of 23–24.9 kg/m^2^ and ≥25 kg/m^2^, respectively.

*Weight bias internalization (WBI)*: The Modified Weight Bias Internalization Scale (WBIS-M) (19) was used to assess the extent to which students blame themselves for stigma and internalize negative weight-based stereotypes. WBIS-M is a modified version of the Weight Bias Internalization Scale (WBIS) (20) and is widely used in community samples (4). This study was translated into Thai by three experts in psychology, psychiatry, and public health, who acted as research counselors, according to a standard translation procedure (21). The WBIS-M is a 10-item scale that is scored on a 7-point Likert scale, ranging from 1 (strongly disagree) to 7 (strongly agree). The overall WBIS-M score is computed by averaging all items, with higher scores indicating higher WBI. We divided the WBIS-M scale into three groups (high, moderate, and low) based on the mean of the scale and one standard deviation (SD) per recommendations by Puhl et al. (22). The mean and SD in this study were 3.39 ±0.86, respectively, so we employed this as our cutoff point. Therefore, a low level of WBI (1 SD below the mean) corresponded to WBIS-M scores ≤2.53, a moderate level corresponded to WBIS-M scores 2.54–4.24, and a high level (1 SD above the mean) corresponded to WBIS-M scores ≥4.25. The scale has strong internal consistency with a Cronbach's alpha of 0.83.

### Outcome Variables

*Quality of life (QOL)*: The primary outcome of interest in this study was QOL, which was assessed using the Thai version of the WHOQOL-BREF (WHOQOL-BREF-THAI), consisting of 26 standard items (23,24). This scale includes four domains, physical health (7 items), psychological health (6 items), social relationships (3 items), and environment (8 items), as well as a self-rating of general QOL (one item) and general satisfaction with health (one item). This self-administered scale used a 5-point response scale ranging from 1 (never) to 5 (always), with higher scores indicating a better QOL. We used recommended cutoffs according to the recommendation of the Thai version of the WHOQOL-BREF by the Department of Mental Health, Ministry of Public Health (24), which has been used in previous studies in Thailand (25–26). The cutoffs for overall QOL scores were classified into two categories: low (26–95 points) and high (96–130 points). Different domains of the QOL were categorized using the following cutoffs for low and high scores: physical health domain: low (7–26 points) or high (27–35 points); psychological health domain: low (6–22 points) or high (23–30 points); social relationships domain: low (3–11 points) or high (12–15 points); and environment domain: low (8–29 points) or high (30–40 points). The scale showed good internal consistency with a Cronbach's alpha of 0.89 for the total QOL scale.

**Statistical analysis**: Descriptive statistics were applied to analyze all variable characteristics. Next, the bivariate odds ratio (OR) was estimated to assess the relationship between the potential predictors and QOL. The adjusted OR was estimated from multivariable logistic regression to evaluate the association between WBI factors and QOL after adjusting for all other predictors. A series model was developed: in Model 1, all socio-demographic factors were added to the model; in Model 2 (the final model), we entered WBI factors into Model 1. All statistical analyses were conducted using SPSS version 20.0 (IBM Corp., Armonk, NY, USA) with a P-value < 0.05 being considered statistically significant.

**Ethics statement**: All participants were informed about the research information, after which they provided written informed consent. The questionnaire was completed by self-report. This research was approved by the Review Ethics Boards of Mahasarakham University (Ethical no. 418-428/2022).

## Results

Over half of the youths were female (51.4%) with a median age of 20 years and a mean BMI of 26.5 kg/m^2^ (SD = 2.5), and about 53.1% reported a monthly household income of 8,000 Thai baht or above (228 US$). More than half (52.5%) of the respondents reported being overweight, and 48.9% reported dissatisfaction with their body image. Approximately 37.8% of respondents reported a high level of WBI (Table 1). Additionally, more than half (57.6%) of the youths reported low levels of overall QOL, followed by low levels in psychological health (57.1%), physical health (56.1%), social relationships (55.2%), and environment domain (53.2%) (Table 2).

**Table 1:** Distribution of socio-demographic factors and WBI factors by overall QOL

Variables	Overall QOL					

	Total (n=667)		Low overall QOL (n=-384)		High overall QOL (n=283)	

	n	%	n	%	n	%
**Socio-demographic factors**						
Age (y)						
< 20	354	53.1	193	50.3	161	56.9
≥ 20	313	46.9	191	49.7	122	43.1
Sex						
Female	343	51.4	204	53.1	139	49.1
Male	324	48.6	180	46.9	144	50.9
Monthly household income (THB)						
< 8000	313	46.9	185	48.2	128	45.2
≥ 8000	354	53.1	199	51.8	155	54.8
Body image satisfaction						
Dissatisfied	326	48.9	208	54.2	118	41.7
Satisfied	341	51.1	176	45.8	165	58.3
Body mass index category (kg/m^2^)						
Overweight (BMI 23 -24.9)	350	52.5	174	45.3	176	62.2
Obese (BMI ≥25)	317	47.5	210	54.7	107	37.8
Body mass index (BMI) (kg/m^2^) (Mean, SD)	26.5	2.5	26.9	2.5	25.9	2.3
**Weight bias internalization (WBI)**						
High	252	37.8	164	42.7	88	31.1
Moderate	218	32.7	132	34.4	86	30.4
Low	197	29.5	88	22.9	109	38.5

Values are presented as number (%) or Mean (S.D.); THB, Thai baht

**Table 2:** The levels of 4 QOL domains, overall QOL and general health

QOL domains	QOL levels			

	Low QOL		High QOL	

	n	%	n	%
Overall QOL	384	57.6	283	42.4
Physical health	374	56.1	293	43.9
Psychological health	381	57.1	286	42.9
Social relationships	368	55.2	299	44.8
Environment	355	53.2	312	46.8

The bivariate analysis of overall QOL revealed that youths with a higher BMI, were dissatisfied with their body image, and had a moderate to high level of WBI were more likely to report low overall QOL. No association between low overall QOL and age, sex, or monthly household income was found. Concerning the multivariate analysis, Model 1 showed that body image dissatisfaction (BD) and BMI were significantly related to low overall QOL. In Model 2, WBI was added to Model 1 and demonstrated moderate to high WBI was strongly associated with worse overall QOL after adjusting for all sociodemographic factors. Moreover, BMI and BD remained significantly related to low overall QOL (Table 3). After examining the four domains of QOL in both models, the results indicated higher BMI and greater BD and WBI were associated with lower levels of QOL in each domain (Table 4).

**Table 3:** Odds ratios and 95% confidence intervals from binary logistic regression for low of quality of life in overall QOL

Variables	Bivariate		Model 1		Model 2 (final model)	

	Unadjusted OR (95%CI)	*P*-value	AOR (95%CI)	*P*-value	AOR (95%CI)	*P*-value
**Socio-demographic factors**						
Age <20 (ref: ≥ 20, y)	0.76 (0.56-1.04)	0.090	0.79 (0.57-1.09)	0.159	0.85 (0.61-1.18)	0.338
Female (ref: male)	1.17 (0.86-1.59)	0.306	1.20 (0.88-1.65)	0.243	1.24 (0.90-1.72)	0.177
Monthly household income < 8000 (ref: ≥ 8000, THB)	1.12 (0.82-1.53)	0.451	1.24 (0.90-1.70)	0.183	1.26 (0.91-1.74)	0.156
Body image	1.65 (1.21-2.25)	0.001	1.59 (1.16-2.19)	0.004	1.69 (1.22-2.34)	0.001
dissatisfaction (BD) (ref: satisfied) BMI	1.18 (1.11-1.26)	<0.001	1.18 (1.10-1.26)	<0.001	1.16 (1.09-1.25)	<0.001
**WBI**						
High (ref: low)	2.31 (1.57-3.38)	<0.001	-	-	2.23 (1.50-3.32)	<0.001
Moderate (ref: low)	1.90 (1.28-2.81)	0.001	-	-	1.94 (1.29-2.91)	0.001

AOR, adjusted odds ratio; CI, confidence interval; ref, reference group; THB, Thai baht

**Table 4:** Odds ratios and 95% confidence intervals from binary logistic regression for low of quality of life in each domain

Variables	Model 1							

	Physical health		Psychological health		Social relationships		Environment	

	AOR (95%CI)	*P*-value	AOR (95%CI)	*P*-value	AOR (95%CI)	*P*-value	AOR (95%CI)	*P*-value
**Socio-demographic factors**								
Age <20 (ref: ≥ 20, y)	0.77 (0.56-1.05)	0.108	0.69 (0.56-0.95)	0.024	0.75 (0.55-1.04)	0.081	0.73 (0.54-1.01)	0.056
Female (ref: male)	1.18 (0.86-1.63)	0.290	1.16 (0.85-1.59)	0.344	1.19 (0.87-1.64)	0.278	1.06 (0.77-1.45)	0.706
Monthly household income < 8000 (ref: ≥ 8000, THB)	1.15 (0.83-1.58)	0.380	1.09 (0.80-1.50)	0.561	1.12 (0.81-1.53)	0.499	1.22 (0.88-1.67)	0.220
BD (ref: satisfied)	1.79 (1.30-2.47)	<0.001	1.60 (1.16-2.19)	0.003	1.69 (1.23-2.33)	<0.001	1.75 (1.28-2.41)	<0.001
BMI	1.19 (1.11-1.27)	<0.001	1.14 (1.07-1.22)	<0.001	1.19 (1.11-1.27)	<0.001	1.18 (1.11-1.27)	<0.001

Variables	Model 2 (final model							

	Physical health		Psychological health		Social relationships		Environment	

	AOR (95%CI)	*P*-value	AOR (95%CI)	*P*-value	AOR (95%CI)	*P*-value	AOR (95%CI)	*P*-value
**Socio-demographic factors**								
Age <20 (ref: ≥ 20, y)	0.82 (0.59-1.14)	0.260	0.75 (0.54-1.03)	0.082	0.81 (0.58-1.12)	0.208	0.78 (0.57-1.09)	0.151
Female (ref: male)	1.22 (0.88-1.69)	0.213	1.20 (0.87-1.66)	0.248	1.24 (0.89-1.70)	0.196	1.09 (0.79-1.51)	0.578
Monthly household income < 8000 (ref: ≥ 8000, THB)	1.17 (0.84-1.62)	0.338	1.12 (0.80-1.54)	0.510	1.13 (0.82-1.57)	0.439	1.24 (0.89-1.71)	0.188
BD (ref: satisfied)	1.91 (1.38-2.64)	<0.001	1.71 (1.23-2.36)	0.001	1.81 (1.31-2.51)	<0.001	1.87 (1.35-2.58)	<0.001
BMI	1.17 (1.10-1.26)	<0.001	1.14 (1.06-1.21)	<0.001	1.18 (1.09-1.26)	<0.001	1.18 (1.10-1.26)	<0.001
**WBI**								
High (ref: low)	2.36 (1.58-3.52)	<0.001	2.54 (1.10-3.78)	<0.001	2.42 (1.62-3.61)	<0.001	2.34 (1.57-3.49)	<0.001
Moderate (ref: low)	1.97 (1.31-2.96)	0.001	2.02 (1.35-3.03)	0.001	2.18 (1.44-3.28)	<0.001	1.98 (1.32-2.98)	0.001

AOR, adjusted odds ratio; CI, confidence interval; ref, reference group; THB, Thai baht

## Discussion

The results demonstrated that greater WBI was significantly associated with a reduction in overall QOL as well as in all four QOL domains (i.e., physical health, psychological health, social relationships, and environment). Consistent with prior studies (5,12), the results indicated that youth with overweight and obesity and who experienced high WBI had poorer QOL. One explanation is that WBI is associated with negative attitudes toward individuals with overweight or obesity, which can negatively affect several aspects of their lives, including physical, psychological, behavioral, and social outcomes (7,12). Studies have also revealed that the negative effects associated with WBI can impair health outcomes and QOL (5,8). For example, WBI can directly affect individuals' emotional states; meaning, those with high WBI may also be more vulnerable to psychological distress, such as depression, anxiety, and low self-esteem, which predicts greater perceived impairment in QOL (4,8). Moreover, WBI negatively affects individuals' eating behaviors (e.g., binge eating, skipping meals, less motivation to diet) and physical activity (e.g., reduction or avoidance of physical activity), which could have an impact on their overall health (4,12,13) and lead to diminished QOL (9,12). Additionally, adolescents with overweight or obesity who had a higher WBI experienced others' negative attitudes and beliefs because of their weight, such as prejudice, negative stereotypes, social rejection, and discrimination (4,6,11). These biased attitudes may lead to social isolation and unfair treatment, which may harm their general physiological, psychological, and social functions (5,7,12). Alternately, WBI may be a psychological stress experience that is stable over time and across important areas of life (4,27); it has been proposed that psychological stress-related WBI may have a significant negative impact on metabolic abnormalities that could potentially lead to poor health (1,2,6) and unhealthy coping behaviors, such as smoking, alcohol use, or binge eating. These impacts could lead to poor QOL (4,12,27). Studies have also shown that individuals with overweight or obesity and WBI have an increased risk of psychological stress (4,27). Thus, higher psychological stress-related WBI plays an important role in the diminished QOL of affected individuals.

In the literature, two self-report measures have been developed to assess internalized weight stigma: the Weight Self-Stigma Questionnaire (WSSQ) and the WBIS (19,28,29). The WSSQ has been adopted and translated into several versions: Iranian (28), Indonesian, (30), and Thai (31). In this study, we measured WBI by using WBIS-M, which is a modified version of the WBIS. This tool is also widely used in community samples and applies to individuals of varying weight statuses (22,28,29). The WBIS has been used in previous studies (28,29,32), similar to our study, the WBIS-M has been used in community samples comprised of individuals with varying weight statuses. Also, some studies have demonstrated that both the WBIS and WBIS-M were valid instruments to assess WBI among adolescents with overweight or obesity and Asian populations (28,29).

Besides, our findings also demonstrated that individuals with a higher BMI had significantly lower levels of overall QOL as well as across all four QOL domains, consistent with previous studies (2,33) that demonstrated QOL was significantly reduced among individuals with a higher BMI. A possible reason is that individuals with a higher BMI, especially those who are overweight or obese, are prone to a high risk of obesity-related health problems, such as muscle pain, articulation pain, and discomfort, as well as greater difficulties in daily activities (1,34). Given that larger bodies require greater physical energy to move, this can influence their physical health. Hence, impaired QOL can be due to the negative health consequences of being overweight or obese (2,35). Additionally, adolescents with overweight or obesity are vulnerable to societal bias and stigma because of their weight, leading to low self-esteem and BD, which can increase psychological health issues (2,6,36). Furthermore, social marginalization or cultural and social pressure based on an ideal body type may lead individuals who believe they do not meet the standard to feel socially rejected, socially undesirable, or discriminated against because of their weight or size. Thus, these negative impacts could contribute to poorer QOL in adolescents (2,8,33).

Moreover, we also found that BD was strongly associated with poor QOL overall as well as across all four QOL domains. According to prior studies (37-39) have reported an association between BD and psychological, social, and physical QOL. A possible reason is that the experience of BD in individuals with overweight or obesity is associated with higher engagement in an emotion regulation process marked by self-criticism and self-judgment, which may lead to self-devaluation due to weight or body size and result in worse QOL (7,37). Additionally, BD has been associated with a range of adverse health outcomes, including eating disorders, depression, low self-esteem, and reduced physical activity. These adverse effects of BD contribute to the impairment of adolescents' emotions and social functioning and lead to poorer QOL (38,39). For example, studies showed that individuals who are dissatisfied with their weight and body are more likely to diet, skip meals, and develop disordered eating. These behaviors may play an important role in unhealthy eating habits and are associated with lower psychological and physical QOL in adolescents (39,40).

This research has some limitations. First, our study was cross-sectional, which precludes the possibility of establishing temporality and causality. Therefore, longitudinal studies to test the causal relationships are needed. Second, the self-reported WBI and QOL may be vulnerable to social desirability bias. To minimize self-report bias, validated and standardized instruments were used. Third, our subjects were university youth with overweight and obesity who had not sought treatment and, therefore, they may have had different experiences with WBI than treatment-seeking adolescents. Thus, caution must be used when generalizing the results to other groups. Fourth, the sample was limited to university students in the northeastern region, which may limit the generalizability of the findings nationwide, but may reflect the situation of overweight and obese youth in the region-based context. Hence, future studies should recruit a nationally representative sample. Despite the limitations, the current study was worthwhile because it was one of the first studies to evaluate WBI and its association with QOL among Thai youths with overweight and obesity. Overall, our findings provide a better understanding of WBI and QOL in the community population and may be useful for health providers or practitioners to be more aware of WBI when dealing with weight issues among youth. Further studies are needed to determine overweight or obesity-related behaviors or adverse impacts of WBI that may contribute to poorer QOL among youth.

In conclusion, higher BMI and greater WBI and BD were strongly associated with poorer QOL overall as well as across the physical health, psychological health, social relationships, and environmental health domains. These findings provide beneficial information for health providers about possible potential factors contributing to impaired QOL in youths with overweight and obesity. This may suggest that the development of interventions to reduce WBI and BD and address problematic BMI may be important for improving QOL overall as well as within specific domains.
